# Applicability and Efficiency of NGS in Routine Diagnosis: In-Depth Performance Analysis of a Complete Workflow for *CFTR* Mutation Analysis

**DOI:** 10.1371/journal.pone.0149426

**Published:** 2016-02-22

**Authors:** Adrien Pagin, Aurore Devos, Martin Figeac, Maryse Truant, Christelle Willoquaux, Franck Broly, Guy Lalau

**Affiliations:** 1 Department of Toxicology and Genopathy, Biology Pathology Center, Lille University Hospital, Lille, France; 2 Functional and Structural Genomic Platform, Lille, France; 3 University of Lille 2, Lille, France; University of Western Australia, AUSTRALIA

## Abstract

**Background:**

Actually, about 2000 sequence variations have been documented in the *CFTR* gene requiring extensive and multi-step genetic testing in the diagnosis of cystic fibrosis and *CFTR*-related disorders. We present a two phases study, with validation and performance monitoring, of a single experiment methodology based on multiplex PCR and high throughput sequencing that allows detection of all variants, including large rearrangements, affecting the coding regions plus three deep intronic loci.

**Methods:**

A total of 340 samples, including 257 patients and 83 previously characterized control samples, were sequenced in 17 MiSeq runs and analyzed with two bioinformatic pipelines in routine diagnostic conditions. We obtained 100% coverage for all the target regions in every tested sample.

**Results:**

We correctly identified all the 87 known variants in the control samples and successfully confirmed the 62 variants identified among the patients without observing false positive results. Large rearrangements were identified in 18/18 control samples. Only 17 patient samples showed false positive signals (6.6%), 12 of which showed a borderline result for a single amplicon. We also demonstrated the ability of the assay to detect allele specific dropout of amplicons when a sequence variation occurs at a primer binding site thus limiting the risk for false negative results.

**Conclusions:**

We described here the first NGS workflow for *CFTR* routine analysis that demonstrated equivalent diagnostic performances compared to Sanger sequencing and multiplex ligation-dependent probe amplification. This study illustrates the advantages of NGS in term of scalability, workload reduction and cost-effectiveness in combination with an improvement of the overall data quality due to the simultaneous detection of SNVs and large rearrangements.

## Introduction

Cystic Fibrosis (CF; MIM #219700) is one of the most frequent autosomal recessive disease, with carrier frequencies among Caucasian populations between 1/20 and 1/40 [1]. CF is a monogenic disorder caused by biallelic germline mutations in the CF transmembrane conductance regulator (*CFTR*/ABCC7; MIM #602421) gene, involved in transmembrane chloride and bicarbonate transport in epithelial cells, mainly affecting lungs, digestive tract, sweat glands and vas deferens in men. Mutations in the *CFTR* gene may also lead to atypical and less severe forms of disease, such as isolated congenital bilateral absence of the vas deferens (CBAVD), chronic pancreatitis or bronchiectasis, regrouped under the term of « *CFTR*-related disorders » (*CFTR*-RD) [2]. The association of two « severe » (i.e. total or almost total loss of function) *CFTR* mutations is generally associated with a « classical » phenotype of CF whereas *CFTR*-RD are caused by combinations of a mild mutation with a severe or with a second mild mutation [3]. Nevertheless, the frontier between CF and *CFTR*-RD may be difficult to establish and the disease is increasingly recognized as a continuum of clinical presentations. The CF or *CFTR*-RD variant classification is not absolute, as some mutations may be associated with a wide range of phenotypical manifestations and be considered as both CF-causing and *CFTR*-RD associated [4]. To date, about 2000 sequence variants have been described in the *CFTR* gene, spread over the 27 coding exons and flanking regions, plus some deep intronic mutations, several large rearrangements and complex alleles [5]. In the context of CF diagnosis, commercial kits permitting the detection of the most frequent CF causing mutations can detect usually around 80–90% of the CF alleles depending on the ethnicity of the patient and the number of mutations tested [6]. However, for *CFTR*-RD diagnosis, the spectrum of mutations is different from CF including a large number of mild mutations that are not included in commercial kits and consequently extensive additional genetic testing is mandatory [7], [8]. Therefore, analytical *CFTR*-RD analysis strategies were developed including a first line test based on commercial kits supplemented with additional analysis using Sanger sequencing, high resolution melting or denaturing high performance liquid chromatography, and copy number variations (CNVs) assessment by multiplex ligation-dependent probe amplification (MLPA) or quantitative multiplex PCR of short fluorescent fragments [2], [4].

In the last decade, next-generation sequencing (NGS) technologies have revolutionized the genetic field with a substancial reduction in cost per base and a considerable enhancement of the sequence generation capacities [9] allowing the analysis of multiples genes concurrently in a short period of time. The benefits of NGS in molecular diagnosis have been widely demonstrated for genetically heterogeneous disorders with the use of gene panels, exome or even whole genome sequencing but may be questioned for monogenic disorder like CF [10], [11]. Indeed, even if NGS have clear practical advantages versus current low-throughput methods its implementation in the routine diagnosis field should be conditional upon the demonstration of at least equivalent analytical performances. For *CFTR* analysis this represents a significant challenge because the gene can be analyzed entirely by Sanger sequencing, which is cumbersome but gives excellent sensitivity and specificity. Moreover, the *CFTR* gene contains several regions that may be critical to analyze by NGS. First, there are several homopolymer (HP) regions located in the coding regions and c.2052del (or 2184delA in the legacy nomenclature), which is one of the 23 most frequent mutations recommended for carrier testing by the American College of Medical Genetics [12], is located in a 7A HP tract that has been previously shown to generate false negative [13] or false positive [14] results in NGS based studies. Secondly, exon 10 (or exon 9 in the legacy numeration) is duplicated in the genome making it potentially difficult to specifically obtain the correct *CFTR* sequence over the duplicated sequence [15]. Finally, intron 9 contains a region composed of a dinucleotidic repeat of 9 to 15 (TG) followed by a 5, 7 or 9 T HP stretch [16]. Therefore, the (TG)mTn locus represents a significant challenge for NGS technologies as well as for interpretation software since it requires precise haplotyping as alleles with 5T are of clinical significance and the number of (TG) associated to 5T alleles modulates splicing of exon 10 [3].

To date, several studies have described NGS protocols for routine molecular diagnosis but several drawbacks remain as the risk of false negative or positive results, or the presence of uncovered targets requesting additional sequencing [14], [17], [18]. The ability of NGS to detect CNVs have been described but there is actually no data on the sensitivity or specificity obtained in large sample cohort that allows for a direct comparison with current techniques.

We present here a two phases evaluation study of a complete workflow based on multiplex PCR amplification for the targeted amplification of *CFTR* followed by MiSeq based NGS and subsequent data analysis. The first phase of the study consists of an initial evaluation of the workflow using previously characterized samples. The second phase comprises a long-term performance monitoring phase in routine diagnostic conditions, particularly regarding CNVs detection.

## Materials and Methods

### Samples analyzed

The validation phase (phase 1) of the study comprised 65 previously characterized DNA samples: 44 positive samples representing a selection of the most frequent *CFTR* pathogenic mutations and/or potentially difficult to detect mutations (InDels, HP and one CNV) (Table 1) and 21 negative samples without pathogenic mutation. In the performance monitoring phase (phase 2) we tested 163 patients referred because of (i) suspicion of an atypical form of CF or *CFTR*-RD (n = 114) or (ii) the presence of fetal bowel hyperechogenicity (n = 46) or (iii) suspicion of a classical form of CF (n = 3). Phase 2 also comprised 94 asymptomatic individuals (partner with at least one CF-causing mutation (n = 34); relative with at least one CF-causing or unknown mutation (n = 51); consanguineous marriage (n = 8)), 18 samples as positive control for point mutations and 3 samples as positive control for CNVs.

**Table 1:** Genotypes tested in validation phase (phase 1).

Patient	Genotype (cDNAposition)	Genotype (Legacy names)	Phenotype
T-01	c.1040G>A	R347H	Carrier
T-04	c.4426C>T	Q1476X	Carrier
T-07	c.1210-34TG[12]T[5]	(TG)12T5	Carrier
T-13	c.1210-34TG[12]T[5]	(TG)12T5	Other
T-15	c.1519_1521del	I507del	CBAVD^2^
T-19	c.1521_1523del	F508del	Carrier
T-20	c.1521_1523del	F508del	Carrier
T-25	c.[1521_1523del];[2052del]	F508del/2184delA^1^	CF
T-26	c.[1652G>A];[2538G>A]	G551D/W648X	CF
T-27	c.[1521_1523del];[2583del]	F508del/2711delT^1^	CF
T-28	c.[1521_1523del];[254G>A]	F508del/G85E	CF
T-29	c.[1521_1523del];[25dup]	F508del/155insG	CF
T-30	c.3376_3381dup	3513insGAAGGA	Carrier
T-31	c.[1521_1523del];[946del]	F508del/1078delT	CF
T-32	c.[2657+5G>A];[1523T>G;3752G>A]	2789+5G>A/S1251N;F508C	CF
T-33	c.[1585-1G>A];[1624G>T]	1717-1G>A/G542X	CF
T-34	c.[1519_1521del];[1210-34TG[13]T[5];3705T>G]	I507del/S1235R;(TG)13T5	Other^3^
T-35	c.[325_327delinsG];[1040G>C]	457TAT>G/R347P	CF
T-36	c.[366T>A];[4374dup]	Y122X/4506insC	CF
T-37	c.2589_2599del	2721del11	Carrier
T-38	c.[2051_2052delinsG];[2657+5G>A]	2183AA>G^1^/2789+5G>A	CF
T-39	c.[870–1113_870-1110del];[3909C>G]	1002-1113delGAAT/N1303K	CF
T-40	c.174_177del	306delTAGA	Carrier
T-41	c.[350G>A];[1000C>T]	R117H/R334W	Newborn Screening^4^
T-42	c.[579+1G>T];[579+1G>T]	711+1G>T/711+1G>T	CF
T-43	c.[1521_1523del];[2052dup]	F508del/2184insA^1^	CF
T-44	c.489+1G>T	621+1G>T	Carrier
T-45	c.[1652G>A];[1210-34TG[11]T[5];3532_3535dup]	G551D/3667ins4;(TG)11T5	CF
T-46	c.[3458T>A];[3889dup]	V1153E/4016insT^1^	CBAVD
T-47	c.[2002C>T;3718-2477C>T]	3849+10kbC>T;R668C	CP
T-48	c.[1521_1523del];[3846G>A]	F508del/W1282X	CF
T-51	c.1210-34TG[12]T[5]	(TG)12T5	CP
T-52	c.[1235del];[1521_1523del]	1367delC/F508del	CF
T-53	c.1364C>A	A455E	Carrier
T-54	c.[2708A>T]	Y903F	Carrier
T-56	c.[617T>G];[868C>T]	L206W/Q290X	CP + CBAVD^5^
T-57	c.1521_1523del	F508del	CP
T-58	c.[164+1G>T];[3140-26A>G]	296+1G>T/3272-26A>G	Bronchiectasis
T-59	c.[1521_1523del];[1657C>T]	F508del/R553X	CF
T-60	c.960A>T	L320F^1^	Carrier
T-61	c.[1521_1523del];[3484C>T]	F508del/R1162X	CF
T-62	c.[262_263del];[1210-34TG[12]T[5]]	394delTT^1^/(TG)12T5	CBAVD
T-63	c.[1585-1G>A];[1210-34TG[13]T[5];3705T>G]	1717-1G>A/S1235R;(TG)13T5	CBAVD
T-64	c.[1521_1523del];[3528del]	F508del/3659delC	CF
T-65	c.-1270-?_11553_?del	CFTRdele1-24	Carrier

Samples without pathogenic variants are not represented in this table.

^1^ Mutation located in a HP stretch (≥ 5 repeats)

^2^Sample from EQA Scheme CF Network 2013

^3^Healthy mother of a CF child with I507del/F508del genotype

^4^Newborn detected in the context of the French newborn screening program with negative sweat test and no symptoms evocative of CF after a 5 years follow-up.

^5^Patient is also heterozygous for the p.Asn34Ser variant of *SPINK1* that predispose to CP.

In total 340 different DNA samples were analyzed, including 316 blood derived DNA samples (extracted with the BACC3 nucleon kit, GE Healthcare Life Sciences, Little Chalfont, United Kingdom), 15 amniotic fluids derived DNA samples (extracted by a phenol-chloroform method), 4 cell cultures DNA derived samples (extracted by a phenol chloroform method) and 5 DNA samples received from other laboratories. Written informed consent was obtained for all the patients and IRB approval was obtained from the “Comité de Protection des Personnes Nord-Ouest IV”.

### Multiplex PCR-based target amplification and resequencing

Target amplification of the 27 exons of *CFTR* with at least 20bp in flanking regions plus three deep intronic mutations (c.870-1113_870-1110delGAAT, c.1680-886A>G and c.3718-2477C>T) was achieved using *CFTR* MASTR v2 kit from Multiplicom (Multiplicom, Niel, Belgium). Briefly, two multiplex PCR reactions containing 30 and 18 amplicons respectively were performed using 50 ng of DNA per PCR reaction. Of these 48 amplicons, 37 are “target amplicons” located on CFTR locus and 11 are “control amplicons” located on chromosomes 6 and 20 for CNVs analysis. Amplicons were tagged in a second PCR with the Molecular Identifiers for Illumina MiSeq kit (Multiplicom) and quality controlled by fluorescent labeling and capillary electrophoresis on an ABI PRISM 3130 (Applied Biosystems, Thermo Fisher Scientific, Waltham, Massachusetts). Per individual, mixing of the two barcoded multiplex PCR products was performed according to a predefined mixing scheme, after which the amplicon libraries were purified two times with Agencourt AMPure XP (Beckman Coulter, Brea, California) on a HAMILTON StarLet 8 platform (Hamilton Robotic, Reno, Nevada) and equimolary pooled. We finally performed quantification and quality control of the library on a 2100 BioAnalyzer instrument (Agilent, Santa Clara, California) and sequencing run were performed on a MiSeq instrument (Illumina, San Diego, California) with a Nano flowcell and the MiSeq Reagent Nano kit V2 with 2x250 bp. A Nano flowcell produced theoretically about 1.10^6^ amplicons clusters per run for a total of 500 Mb of data for the 2x250 bp chemistry. Each cluster produced a first read R1 of 250 bp starting from the beginning of the F primer and a second read R2 starting from the beginning of the R primer. In each run we have included at least one positive control with known SNVs/InDels or CNVs and a blank.

### Data analysis

Data was analyzed in parallel with two independent bioinformatic pipelines against NM_000492.3. The first pipeline involved alignment, coverage analysis and variant calling with the SeqNext module of the SeqPilot software v4.1.2 (JSI Medical Systems, Ettenheim, Germany). Regions of interest (ROIs) plus exact amplicons positions were defined on the SeqNext software using the definition file provided by Multiplicom, specially dedicated for SeqNext setup, allowing the exclusion of the primers from the analysis and bases with a quality score <20 were filtered-out. The second pipeline included MiSeq Reporter software v2.3.32, which used BWA [19] for alignment and GATK [20] for InDels re alignment and variant calling (exact coordinates of the ROIs are available in the supplementary material). The Variant Allele Frequency (VAF) threshold for variant detection was set to 10% for both pipelines. Sequencing events with the following characteristics were considered as recurrent sequencing artifacts: i) presence in most patients of a run with VAF inferior to 30% ii) not present in both R1 and R2 directions iii) not a known pathogenic or frequent variant.

CNVs analysis was also performed with the SeqNext module. A relative PCR coverage (RPC_CNV_) ratio is calculated for each target amplicon taking into account the coverage variations between target and control amplicons and the comparison of each sample with the totality of the other samples sequenced in the same run with the exception of the positive CNVs control (detailed information is available on http://www.jsi-medisys.de/). Amplicons RPC_CNV_ was calculated independently for the two multiplex PCRs and thresholds were set to 0.7 for deletions and 1.3 for duplications.

We also developed an in-house script to specifically analyze the (TG)mTn locus. The principle is to count in the unaligned reads every possible combination of (TG)mTn plus anchor bases on 5’ and 3’ of the locus, and to consider the two most frequent combinations as the two alleles. If a combination is present at a frequency superior to 50% of all reads, then the patient is considered as homozygous for this combination (bash script available upon request).

### Variant confirmation and annotation

Potentially pathogenic variants were confirmed by Sanger sequencing or MLPA (SALSA MLPA P091 CFTR kit, MRC Holland, Amsterdam, Netherlands). Sequence variations are annotated according the recommendations of the Human Genome Variation Society in accordance with the *CFTR* gene numbering in which nucleotide +1 in the coding DNA reference sequence (GenBank NM_00492.3) corresponds to the A of the ATG translation initiation codon. For convenience, the denomination with the legacy nomenclature [5] was added in the tables or in parentheses in the text after the HGVS nucleotidic denomination.

### Design of the study

In the phase 1 of the study we assessed the completeness of the *CFTR* regions in the design of the *CFTR* MASTR v2 kit and the ability of the total workflow to detect all kind of *CFTR* variants. Furthermore, we also compared the results obtained with the two bioinformatic pipelines, in order to validate the analysis settings based on the available variant information. In phase 2 we evaluated the robustness of the workflow in routine diagnostic conditions with a larger number of samples and focused on the homogeneity of the coverage between amplicons, the false positive rate and the accuracy of the CNVs detection.

## Results

### Phase 1: Validation phase

The 65 phase 1 samples were sequenced in two MiSeq Nano flowcell runs yielding 499 and 565 Megabases (Mb) respectively. Coverage analysis with both analysis pipelines indicated that 100% of the ROIs were covered. The average read depth across all 65 samples was 607.

#### Variant detection

All known pathogenic variants (67 heterozygous and one homozygous) (Table 1) as well as all frequent neutral variants (data not shown) were identified by both analysis pipelines. We didn’t identify any undocumented and potentially pathogenic variants and no false positive results were observed.

The RPC_CNV_ based CNVs analysis correctly detected the entire *CFTR* heterozygous deletion present in the control sample with all *CFTR* amplicons showing a RPC_CNV_ below the 0.7 threshold (value between 0.440 and 0.678). We evaluated the performance of our in-house script to correctly determine the (TG)mTn haplotype on 30 samples of the phase 1 representing different possibilities of clinically relevant alleles ((TG)11T5 n = 1; (TG)12T5 n = 4; (TG)13T5 n = 2) and non-pathogenic alleles ((TG)10T7 n = 16; (TG)11T7 n = 25; (TG)12T7 n = 1; (TG)9T9 n = 1; (TG)10T9 n = 10). Our in-house script correctly determined all (TG)mTn alleles in a blinded analysis by two independent operators (Table 2).

**Table 2:** (TG)mTn haplotyping with an in-house script.

		Percentage of reads attributed to each (TG)mTn combination								
Patient	(TG)mTn haplotype	(TG)11T5	(TG)12T5	(TG)13T5	(TG)10T7	(TG)11T7	(TG)12T7	(TG)9T9	(TG)10T9	Others
T-42	(TG)11T7/(TG)11T7	0	1	0	24	**69**	4	4	0	2
T-63	(TG)13T5/(TG)10T7	7	16	**28**	**46**	1	0	0	0	2
T-51	(TG)12T5/(TG)11T7	14	**32**	3	14	**33**	1	1	0	6
T-62	(TG)12T5/(TG)10T9	16	**33**	1	1	1	0	9	**30**	9

Table 2 illustrates the results obtained with our in-house script for different combination of (TG)mTn alleles. Values between 25 and 50 indicate heterozygosity and values above 50 indicate homozygosity. Usually about 10-30% of the reads lacked one or two TG repeat but the number of T repeat is correct. The script detects all the possible combinations of (TG)mTn but for a reason of clarity we only present results for combinations detected at > 5%.

#### Bioinformatic pipelines

**Fig 1:**
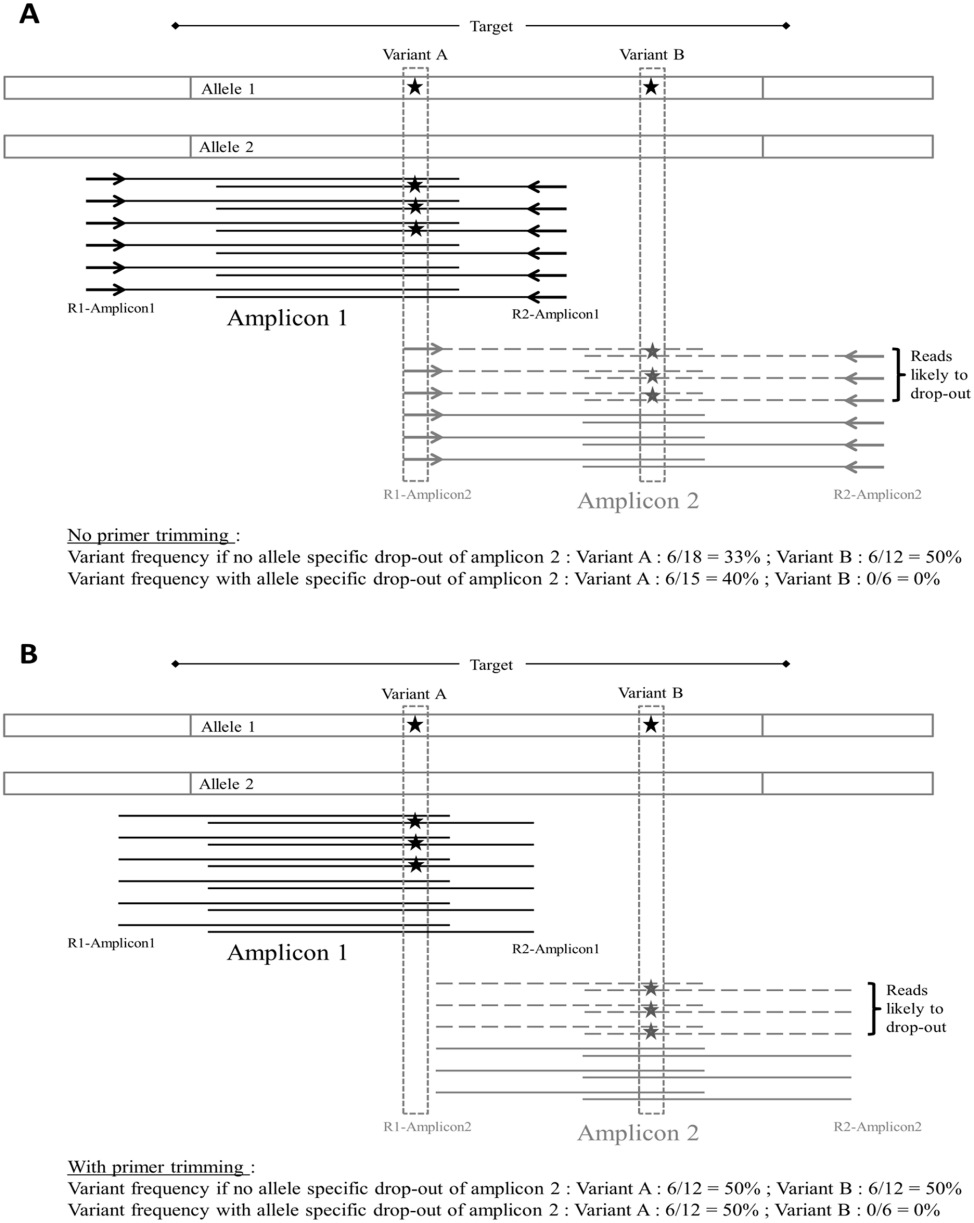
Impact of primer trimming on variant calling. (A) Overestimation of WT allele at variant A locus due to the presence of the primers sequence in the sequencing reads of Amplicon 2 as observed for c.1000C>T (the same principle applies to c.2583del and c.2589_2599del, which are located under the R2 primer of Amplicon 1). When no allele drop-out occurred, variant A is present in 3/12 reads in R1 direction (3/6 from Amplicon1 and 0/6 from Amplicon2) and 3/6 reads in R2 direction for Amplicon1 for a total VAF of 6/18 = 33%. There is a risk of false negative for variant A if the depth of coverage of Amplicon2 is superior to Amplicon1, resulting in a VAF under the detection threshold. In the case of an allele drop-out of Amplicon 2 due to the presence of variant A, then variant A is present in 3/9 reads in R1 direction (3/6 from Amplicon1 and 0/3 from Amplicon2) and 3/6 reads in R2 direction for Amplicon1 for a total VAF of 6/15 = 40%. In this case there is also a risk of false negative if a second variant is associated in cis with variant A (as illustrated by variant B). (B) Impact of primer trimming to limit the risk of false negative results. When primer sequences are removed from the reads, the VAF for variant A is 50% independently of the risk of allele drop-out. In the case of a complex allele involving variant A and B, the risk of false negative result for variant B due to allele drop-out of Amplicon 2 is still present and can be addressed by concomitant CNVs detection followed by Sanger sequencing with a different primer set.

In order to compare the 2 analysis pipelines for coverage analysis and variant calling, we calculated for the 68 variants identified the ratio between SeqNext and MiSeq Reporter values for coverage (COV_SN/MR_) and VAF (VAF_SN/MR_). For coverage values, mean COV_SN/MR_ was 0.997 with values between 0.90 and 1.10 for 64 of the 68 variants. For VAF values, mean VAF_SN/MR_ was 1.015 with values between 0.90 and 1.10 for 64 of the 68 variants. Four variants (c.1000C>T (R334W), c.1235del (1367delC), c.2583del (2711delT) and c.2589_2599del (2721del11)) felt outside of the 0.90–1.10 range for both COV_SN/MR_ and VAF_SN/MR_ (Table 3). Three variants (c.1000C>T, c.2583del and c.2589_2599del) co-located with a primer hybridization site for one of two overlapping amplicons. CNVs analysis of the redundant amplicons showed a single amplicon deletion of amplicons with ratios of 0.53, 0.62 and 0.60, respectively, indicating an allele drop-out (ADO). In those three cases, the differences observed between SeqNext and MiSeq Reporter values are related to the primer trimming, which was achieved with SeqNext but not with the MiSeq Reporter (Fig 1 and Table 3). Consequently, an excess of wild type genotype reads is observed with MiSeq Reporter when a variant is present at a primer hybridization locus (Table 3).

**Table 3:** Variant presenting coverage and variant frequency discrepancies between SeqNext and MiSeq Reporter.

	MiSeq Reporter						SeqNext						Comparison	
	R1 WT	R1 mut.	R2 WT	R2 mut.	Cov.	VAF	R1 WT	R1 mut.	R2 WT	R2 mut.	Cov.	VAF	COV_SN/MR_	VAF _SN/MR_
c.1000C>T^1^	**276**	53	71	52	452	23.2	**70**	53	70	52	245	42.9	0.54	1.85
c.1235del	210	**136**	213	207	766	44.8	217	**216**	220	219	872	49.9	1.14	1.14
c.2583del ^1^	481	439	**551**	1	1472	29.9	550	373	**0**	0	923	40.4	0.63	1.35
c.2589_2599del ^1^	220	156	**61**	0	437	35.7	225	155	**0**	0	380	40.8	0.87	1.14

Number of mutant (mut.) and WT reads in each direction, total coverage (Cov.) and VAF for variants presenting discrepancies between the two analysis pipelines using different primer trimming settings. Read count values showing discrepancies are in bold.

^1^Variant located at a primer hybridization site.

### Phase 2: Performance monitoring phase

As all phase 1 variants were identified with both analysis pipelines without false positive results, the 10% VAF threshold was conserved for variant detection in phase 2.

#### Depth of coverage analysis

**Fig 2:**
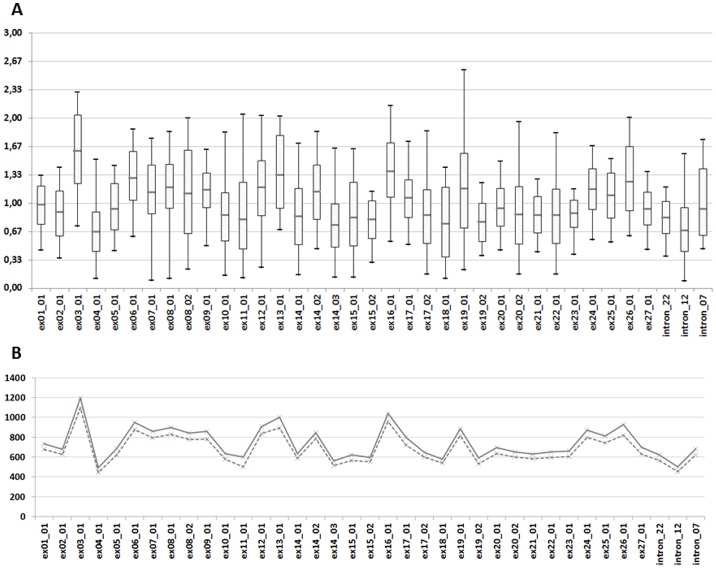
Depth of coverage analysis for phase 2 samples. The coverage analysis is based on the number of R1 reads counted for each amplicon with MiSeq Reporter. Values were calculated for the 275 samples tested in phase 2 (CNVs positive controls were excluded). (A) Box plot diagram of the RPC_COV_. Box plots show mean (horizontal grey line), 95^th^ percentile values (box outline) and minimal/maximal values (whiskers). (B) Mean (solid line) and median (dotted line) depth of coverage values by amplicon.

The 278 DNA samples from the phase 2 were sequenced in 15 Miseq runs with an average yield of 499 Mb (min = 246 and max = 637). To evaluate the uniformity of the amplicon coverage we calculated a relative PCR coverage (RPC_COV_) for every amplicon by dividing the number of reads per amplicon by the average read depth of the sample (RPC_COV_ = number of reads per amplicon / mean coverage of the sample). RPC_COV_ was calculated for each amplicon in each sample of the phase 2 (n = 275; positive CNV control were excluded from the analysis) (Fig 2A). Overall we observed for *CFTR* amplicons that 99.7% of the RPC_COV_ values were comprised between 0.33 and 3. Only 29 (or 0.28%) amplicons in all phase 2 samples were covered with a depth inferior to one third of the mean depth of the patient (RPC_COV_ below 0.33). Those 29 amplicons were spread over 6 samples representing 2.2% of the tested population. Overall, the average read depth across all 278 samples was 752 with mean depth between 186 and 2032 per patient. Mean and median depth of coverage values are shown for each amplicon on Fig 2B. The lowest depth observed for an amplicon was 48 and the highest was 4526. Of note, for 9 of the 15 MiSeq runs, *CFTR* assays were multiplexed with other genes assays resulting in a reduction of the number of reads attributed to *CFTR* samples (about 30% of the reads for those 9 runs were attributed to other genes assays).

#### Variant detection

There were 63 heterozygous and 1 homozygous variants identified by both analysis pipelines among the 257 patients. Additionally, the 18 heterozygous and 1 homozygous variants of the positive controls were also identified by both analysis pipelines (Table 4 and S1 Table). No false positive results were observed.

**Table 4:** Variants identified in the performance monitoring phase (Phase 2).

**Patients (n = 257)**	
**SNVs/InDels**	**c.91C>T** (R31C), **c.220C>T** (R74W), **c.350G>A** (R117H; n = 4), **c.472G>A** (S158G), **c.508C>T** (R170C), **c.650G>A** (E217G), **c.674G>A** (C225Y; n = 2), **c.721G>T** (G241W), **c.870-1113_870-1110delGAAT** (1002-1113delGAAT; n = 2), **c.1117-5A>G** (1249-5A>G), **c.1130dup** (1259insA), **c.1327G>T** (D443Y), **c.1364C>A** (A455E; n = 2), **c.1393-1G>A** (1525-1G>A), **c.1521_1523del** (F508del; n = 10 with one homozygous patient), **c.1624G>T** (G542X), **c.1696G>A** (A566T), **c.1727G>C** (G576A; n = 4), **c.1684G>A** (V562I), **c.2002C>T** (R668C; n = 2), **c.2039del** (2171delC), **c.2210C>T** (S737F; n = 2), **c.2450G>T** (G817V), **c.2490+4_2490+7del** (2622+4delGGTA), **c.2735C>G** (S912W), **c.2743G>C** (V915L), **c.2900T>C** (L967S), **c.2991G>C** (L997F), **c.3209G>A** (R1070Q), **c.3208C>T** (R1070W), **c.3322G>C** (V1108L), **c.3415A>G** (I1139V), **c.3485G>T** (R1162L; n = 2), **c.3705T>G** (S1235R; n = 4), **c.3808G>A** (D1270N), **c.3909C>G** (N1303K), **c.3935A>G** (D1312G), **c.4243-5C>T** (4375-5C>T), **c.4433C>G** (T1478R)
**(TG)mTn**	**c.1210-34TG[11]T[5]**^1^ ((TG)11T5; n = 12), **c.1210-34TG[12]T[5]**^1^ ((TG)12T5; n = 10)
**Positive Controls (n = 21)**	
**SNVs/InDels**	**c.350G>A** (R117H; n = 2), **c.1000C>T** (R334W), **c.1117-5A>G** (1249-5A>G), **c.1521_1523del** (F508del; n = 11 with one homozygous control), **c.1585-1G>A** (1717-1G>A), **c.2039del** (2171delC), **c.2052dup**^1^ (2184insA), **c.2589_2599del** (2721del11), **c.3468+1G>T** (3600+1G>T)
**CNVs**	**c.54-5940_273+10250del** (CFTRdele2_3; n = 14), **c.274-?_1679+?dup** (CFTRdup4_10; n = 2), **c.-1270-?_11553_?del** (CFTRdele1_24)
**(TG)mTn**	**c.1210-34TG[11]T[6]**^1^ ((TG)11T6)

^1^ Mutation located in a HP stretch (≥ 5 repeats).

#### CNVs detection

**Fig 3:**
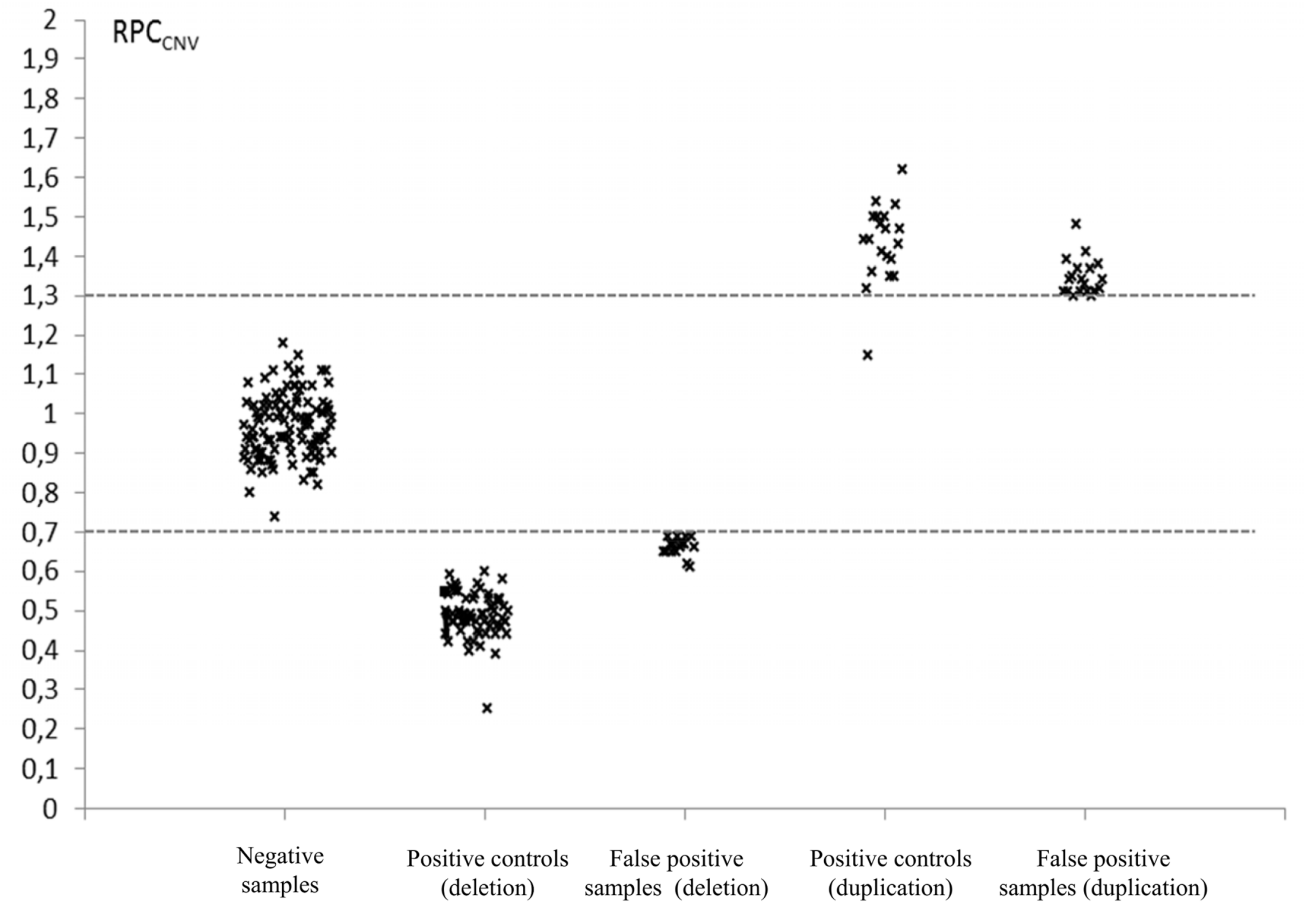
CNVs analysis of Phase 2 samples. RPC_CNV_ for (1) every amplicon of 3 negative samples verified by MLPA, (2) deleted amplicons for CFTRdele2_3 (n = 14) and CFTRdele1_24 (n = 1) positive controls, (3) apparently deleted amplicons in false positive samples (n = 6 samples for 15 amplicons), (4) duplicated amplicons for CFTRdup4_10 controls (n = 2) and (5) apparently duplicated amplicons in false positive samples (n = 14 samples for 20 amplicons). Three samples showed false positive signals for both deletion and amplification. Dot lines indicate thresholds for deletion (0.7) and duplication (1.3).

CNV detection was performed for every sample of the phase 2, and 3 positive controls for CFTRdele1_24, CFTRdup4_10 and CFTRdele2_3 were added in one, two and 14 runs, respectively. 100% of the positive controls were correctly detected with every deleted or duplicated exons showing a RPC_CNV_ < 0.7 or >1.3, respectively, with the exception of a single amplicon targeting exon 7 that showed a RPC_CNV_ = 1.15 for a patient with CFTRdup4_10. For positive CNVs controls, mean RPC_CNV_ was 0.489 for amplicons targeting deleted exons and 1.432 for amplicons targeting duplicated exons. In the 257 patients analyzed, 240 showed strictly normal CNVs profiles (RPC_CNV_ in 0.7–1.3 range), 12 were abnormal for a single amplicon and 5 presented several amplicons outside of the expected range. The RPC_CNV_ values of the 17 samples with one or more apparently deleted or duplicated amplicon were distinct from the normal CNV profile cluster with mean RPC_CNV_ = 0.662 for deleted amplicons and 1.344 for duplicated amplicons, indicating borderline results when compared with positive controls, especially for deleted amplicons (Fig 3). All 17 were identified as false positive by MLPA verification. We additionally verified by MLPA 20 randomly selected samples with normal CNV profiles without identifying any false negative result.

#### (TG)mTn haplotyping

For the (TG)mTn region, we verified by Sanger Sequencing every sample with a 5T allele as well as samples with an uncommon allele. Among the 257 patients, we identified with our in house script 12 samples with (TG)11T5 and 10 with (TG)12T5, plus one sample with a rare (TG)8T7 allele. We obtained 100% concordance between the NGS data analyzed with our script and Sanger Sequencing for these alleles as well as for the second common allele associated. We also tested a positive control with a rare (TG)11T6 sequence that was correctly detected.

#### Reproducibility

Adding the CFTRdele2_3 positive control in 14 different runs also allowed us to evaluate the reproducibility of our technique for the three different types of variant (sequence variations, (TG)mTn and CNVs). The CFTRdele2_3 was successfully identified in 14/14 runs, all the non-pathogenic variants (c.744-9_744-6del, p.Val470Met, c.869+11C>T, c.1680 870T>A and c.3367+45T>C) were identified in 14/14 runs by both analysis pipelines and our in house script identified a (TG)11T7/(TG)10T9 haplotype in the 14 runs.

## Discussion

The utility of NGS technologies is clearly established in situations requiring to sequence large amount of kilobases or megabases, like in the case of genetically heterogeneous disorders or to permit the analysis of a complete gene locus, including the intronic regions, as recently demonstrated for *CFTR* [21], [22]. In the case of *CFTR* routine analysis, where classical sequencing methods are well established, NGS may represent a more straightforward and cost-efficient alternative but its implementation should be conditioned upon the demonstration of at least equivalent analytical performances. Published NGS protocols for *CFTR* analysis have demonstrated satisfactory overall results but some constant problems remain like the risk of false negative results or the presence of uncovered targets requiring additional Sanger sequencing [13], [14], [17], [18]. Certain studies have demonstrated that the detection of CNVs was achievable with targeted NGS protocols [14], [18] but lack data to evaluate the sensitivity or specificity and hence to evaluate its applicability in routine diagnosis. We present here a comprehensive evaluation on 340 samples of a NGS-based workflow for *CFTR* analysis that was developed for use in routine diagnostic conditions with short turn-around time (TAT) and to permit the detection of all kind of variants, including large genomic rearrangements, targeting all 27 coding exons and the flanking intronic sequences plus three deep intronic loci. We reported 100% coverage of every sample tested with a variant detection rate of 100% on control samples, which were enriched with the most frequent *CFTR* pathogenic mutations and/or potentially difficult to detect mutations (InDels, HP and CNV) (Table 1). We also provided detailed data regarding CNVs detection and reported a 100% detection rate in positive controls with a low rate of false positive signals, supporting its use in routine diagnostic.

The sequencing data was analyzed by two independent bioinformatic pipelines that both showed a 100% detection rate and 100% specificity for SNVs and InDels. Results were very similar with the advantage for SeqNext to allow for precise primer trimming, thus avoiding the overestimation of the WT nucleotide whenever a variant is present at a primer site and limiting the risk of false negative. Despite the apparent redundancy of the two analyses, we didn't consider choosing for a particular tested pipeline for several reasons. First, the use of two independent pipelines using different alignment and variant calling algorithms is considered as a security measure as it is not possible to prove that a given software will ensure perfect variant calling for any virtually possible events affecting the gene sequence. Secondly, the two pipelines offer complementary characteristics with MiSeq Reporter's analysis being fully automated whereas SeqNext requires more manual intervention but provides a user friendly and precise visualization of the data. Finally, the combined use of the two pipelines also limits the risk of erroneous result due to human error in the bioinformatic steps.

For the (TG)mTn haplotype determination we developed a dedicated in-house script that basically count every possible combination of (TG)mTn in the unaligned reads because the repetitiveness and the polymorphism of this particular nucleotidic sequence produce recurrent artifacts with both SeqNext and MiSeq Reporter. The concordance between our in-house script and Sanger sequencing was 100% for both (TG)mTn alleles on a total of 55 samples. We observed that usually about 70% of the sequencing reads are bearing the exact (TG)mTn sequence and 30% are counted as (TG)m-1Tn and (TG)m-2Tn (Table 3). This “slippage” is similar to what is observed using fragment analysis and makes us hypothesized that at least part of the observed errors are introduced during the amplification steps, before the sequencing on MiSeq (see supplementary data). Our results also indicate a good specificity of the assay for targeting the exon 10 of *CFTR* which possessed pseudo-exons located on chromosomes 9,12 and 20 [15], as all the known variants of exon 10 and flanking regions included in the study were correctly detected ((TG)mTn, c.1235del (1367delC), c.1327G>T (D443Y) and c.1364C>A (A455E)) with no false positive results. We analyzed exon 10 specificity in detail by aligning the sequencing reads to the human genome. This analysis showed that all obtained exon 10 sequences aligned to *CFTR* and not to the duplicated position.

The ability of the assay to detect CNVs was also excellent with a 100% detection rate (18/18) of the positive controls. Among the 257 patients tested in the phase 2, we observed 240 normal CNVs profiles (93.4%) and 17 samples (6.6%) with an apparent deletion or duplication showing borderline RPC_CNV_ values of at least one amplicon that was not confirmed by MLPA. This means that no CNVs were identified besides the positive controls during the study. This is coherent with the low frequency of large rearrangements in *CFTR*-RD patients which represent the majority of the tested samples [23]. The majority of the false positive profiles calls demonstrated borderline RPC_CNV_, especially for deletion (Fig 3), and none displayed two consecutive affected amplicons, thus limiting the risk of misinterpretation. Nevertheless, CNVs affecting a unique exon have been documented in *CFTR* [24], [25] and some of those samples may require verification by another quantitative method that may considerably delayed the result report. This problem could have been minimized in the design of the multiplex PCRs by systematically targeting all exons, including the smallest ones, by at least two amplicons to facilitate the interpretation of CNVs analysis, but with the counterpart to potentially compromise the balance between amplicons and to reduce the number of samples that can be simultaneously analyzed per run.

In the phase 2 of the study, we demonstrated a remarkable coverage homogeneity with 99.7% of the amplicons presenting a coverage higher than 0.33 of the mean per sample and a minimal read depth of 48 for the lowest amplicon, which means no need for additional Sanger verification due to uncovered target. As a consequence, if we set the minimum read depth request to 40 we only need a mean coverage per patient of 120 to reach a depth coverage of 40 for 99.7% of the target regions. With the design containing 48 amplicons, the total number of reads requested by patient would then be 5760 and the theoretical number of patient that can be sequenced per run is 173 (considering that the mean yield for a Nano flowcell is 1x10^6^ clusters of reads per run). However, this number remains theoretical as other factors have to be taken into account (e.g. the quality of the quantification of each sample PCRs products before pooling) and because most diagnostic laboratories cannot use the full sequencing capacity because of TAT considerations. In order to optimize the cost effectiveness of the Miseq runs, we choosed to perform a run every 10–15 days containing approximately 20 *CFTR* samples together with other targeted gene assays. This has been done for 9 of the 15 runs of the phase 2 without any significative negative impact on the *CFTR* assay as shown by the depth of coverage homogeneity. With this practical organization the TAT for *CFTR* analysis is between 1 and 4 weeks depending on the timing of sample arrival and the need for Sanger or MLPA verification with a total cost per patient of approximately 130 euros.

This new routine NGS approach has considerably changed the organization of our *CFTR* diagnostic workflow mainly by introducing batch processing samples and the reduction of the number of molecular technique required to perform a complete analysis. With almost one year of experience, we have observed a significant reduction of the technical workload, as the complete "wet lab" workflow requires only one technician (1,5 days) before starting the 24 hours sequencing run. Also, the obtained reduction in data analysis is significant with the complete "dry lab" part of the workflow that can be performed in half a day by two persons in parallel. Overall, the transition from Sanger sequencing to NGS allowed us to slightly decrease the mean TAT but batch processing limits the prioritization of urgent samples. The implementation of NGS has revealed several advantages in comparison to the classical scanning or sequencing techniques. The possibility to amplify in two reactions the complete coding regions of *CFTR* instead of the former 31 individual PCRs and 62 sequencing experiments significantly reduces the risk of human error, as does the semi-automation of the workflow and the barcoding of the PCR products. To monitor for pre-barcoding inter-sample contamination, we decided to set the VAF detection threshold to 10%. The quantitative aspect of the technology thus enables the detection of low frequency variants that can identify potential cross contamination between samples. We didn’t detect cross contamination in this study as all the low VAF events detected may be considered as obvious recurrent sequencing errors. In our mind, the main technical progress introduced by this technique is the possibility to combine an accurate detection of SNVs, InDels and CNVs in the same experiment not only for practical reasons but also because it prevents the risk of false negative related to ADO. In our study, we detected three cases of drop-out of an amplicon due to the presence of a sequence variation underneath a primer binding site which prevents hybridization of the primer. In case of ADO, if a second variant is present in the same allele within the affected amplicon it will go undetected (Fig 1). When a potential ADO is detected by CNVs analysis (i.e. isolated deletion of an amplicon), sequencing of the concerned exon by Sanger method with different primers permits to avoid the risk of false negatives and to confirm the presence of the variant at the primer binding site. Theoretically, there is also a risk of “bioinformatic ADO” in the analysis of NGS data, when a variant changes considerably the sequence of a gene and prevents the correct alignment of the reads. We hypothesize that our workflow must be able to detect such a phenomenon because it will appear as a single amplicon deletion and should be detected by Sanger verification.

In conclusion, we demonstrated that multiplex PCR based amplification in combination with MiSeq sequencing can advantageously replace conventional diagnostic methods and increased both the quantity and quality of the final molecular analysis, by simultaneously detecting punctual variants and CNVs. We also provided original data on CNVs detection on a large numbers of experiments supporting its applicability in routine diagnostic laboratories. Overall, the workflow described here demonstrated equivalent diagnostic performances between multiplex PCR based targeted NGS analysis and Sanger sequencing and MLPA with the added advantage of ADO detection. Finally, we demonstrated that NGS can remain cost effective in laboratories with small or medium throughput by the simultaneous implementation of different gene assays.

## Supporting Information

S1 Appendix Positions (Hg19) of the Regions Of Interest (ROIs) for variant calling. *CFTR* genomic positions (27 exons plus three deep-intronic loci) considered for variant calling for both bioinformatic pipelines used in the study.
(DOC)

S1 Fig Quality control profiles of barcoded amplicons after fluorescent labeling and capillary electrophoresis. (A) Amplicons targeting exons 17, 4, 10 and 14 of CFTR. The (TG)mTn is located upstream of exon 10 and amplified with the 10_01 amplicon. Amplicons 17_02, 4_01 and 14_03 appeared mainly as a single peak whereas amplicon 10_01 shows a slippage with secondary peaks at n-1 and n-2 size and very weak peaks at n-3 and n-4. This profile makes us hypothesized that at least a part of the sequence errors observed on (TG)mTn with our in house script is introduced in the amplification steps before sequencing on MiSeq. (B) Profile of amplicon 10_01 for a sample compound heterozygous for (TG)11T5 and (TG)11T7. (C) Profile of amplicon 10_01 for a sample homozygous for (TG)11T7. (D) Amplicons targeting exons 19 of *CFTR* and controls regions 7, 8 and 9 on chromosome 6 and 20. Amplicon 19_01 also exhibit a slippage which may be related to the presence of a HP stretch of 13 A on the amplicon that generates a high rate of error of the polymerase. This stretch is located 93 nucleotides upstream of exon 19 and was excluded of the ROIs because it generated a high rate of false positive InDels. This support the hypothesis that a significant proportion of the sequencing errors observed in repetitive regions is introduced by PCRs independently of the NGS platform used.
(DOC)

S1 Table Genotypes identified in the performance monitoring phase (phase 2). Samples without pathogenic variants are not represented in this table. Patients with the “Atypical CF” phenotype were referred in the context of respiratory symptoms (e.g. nasal polyposis, chronic rhino-sinusitis, chronic cough) or digestive manifestations (malabsorption) and present intermediary or positive sweat test values. Patients with the “Carrier” phenotype do not present any clinical signs evocative of CF or *CFTR*-RD (healthy parents of a foetus with hyperechogenic bowels are included in this group). ^1^ Healthy father of a CF child. ^2^ This patient was refered because of positive family history and is fertile and asymptomatic.
(DOC)
