# Supplementary material for: Applicability and Efficiency of NGS in Routine Diagnosis: In-Depth Performance Analysis of a Complete Workflow for CFTR Mutation Analysis
Source: PLoS One. 2016 Feb 22;11(2):e0149426. doi: 10.1371/journal.pone.0149426 (PMC4762772; doi:10.1371/journal.pone.0149426)
Supplement: S1 Appendix — CFTR genomic positions (27 exons plus three deep-intronic loci) considered for variant calling for both bioinformatic pipelines used in the study. (DOC) [file pone.0149426.s001.doc]

**S1 Appendix.**

Positions (Hg19) of the Regions Of Interest (ROIs) for variant calling

| chr7 | 117120041 | 117120308 | CFTR-E01 |
| --- | --- | --- | --- |
| chr7 | 117144246 | 117144508 | CFTR-E02 |
| chr7 | 117149066 | 117149248 | CFTR-E03 |
| chr7 | 117170909 | 117171224 | CFTR-E04 |
| chr7 | 117174158 | 117174461 | CFTR-E05 |
| chr7 | 117175206 | 117175511 | CFTR-E06 |
| chr7 | 117176507 | 117176795 | CFTR-E07 |
| chr7 | 117179013 | 117179191 | CFTR-1113delTAAG |
| chr7 | 117180112 | 117180556 | CFTR-E08 |
| chr7 | 117181900 | 117182183 | CFTR-E09 |
| chr7 | 117188612 | 117188928 | CFTR-E10 |
| chr7 | 117199461 | 117199743 | CFTR-E11 |
| chr7 | 117227675 | 117227918 | CFTR-E12 |
| chr7 | 117229455 | 117229590 | CFTR-1811+16kb |
| chr7 | 117230343 | 117230522 | CFTR-E13 |
| chr7 | 117231937 | 117232736 | CFTR-E14 |
| chr7 | 117234829 | 117235331 | CFTR-E15 |
| chr7 | 117242838 | 117243005 | CFTR-E16 |
| chr7 | 117243492 | 117243887 | CFTR-E17 |
| chr7 | 117246587 | 117246880 | CFTR-E18 |
| chr7 | 117250493 | 117250911 | CFTR-E19 |
| chr7 | 117251514 | 117251990 | CFTR-E20 |
| chr7 | 117254605 | 117254918 | CFTR-E21 |
| chr7 | 117267555 | 117267865 | CFTR-E22 |
| chr7 | 117279950 | 117280100 | CFTR-3849+10kb |
| chr7 | 117282401 | 117282711 | CFTR-E23 |
| chr7 | 117292792 | 117293020 | CFTR-E24 |
| chr7 | 117304713 | 117304951 | CFTR-E25 |
| chr7 | 117305373 | 117305644 | CFTR-E26 |
| chr7 | 117306916 | 117307193 | CFTR-E27 |
