# Supplementary material for: Applicability and Efficiency of NGS in Routine Diagnosis: In-Depth Performance Analysis of a Complete Workflow for CFTR Mutation Analysis
Source: PLoS One. 2016 Feb 22;11(2):e0149426. doi: 10.1371/journal.pone.0149426 (PMC4762772; doi:10.1371/journal.pone.0149426)

**S1 Figure**

**Quality control profiles of barcoded amplicons after fluorescent labeling and capillary electrophoresis**

**A**


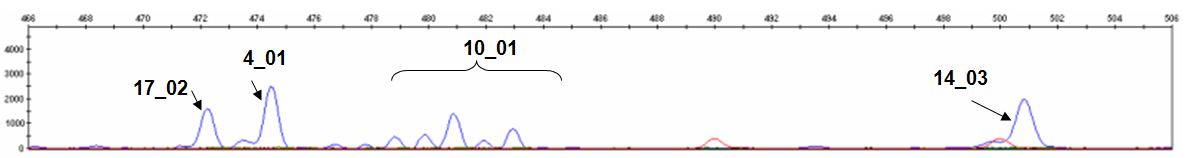


**B**

**C**

**D**


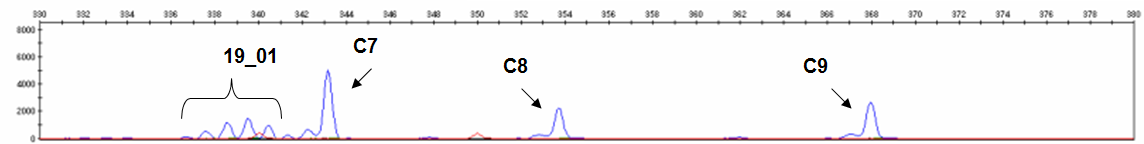

Supplement: S1 Fig — (A) Amplicons targeting exons 17, 4, 10 and 14 of CFTR. The (TG)mTn is located upstream of exon 10 and amplified with the 10_01 amplicon. Amplicons 17_02, 4_01 and 14_03 appeared mainly as a single peak whereas amplicon 10_01 shows a slippage with secondary peaks at n-1 and n-2 size and very weak peaks at n-3 and n-4. This profile makes us hypothesized that at least a part of the sequence errors observed on (TG)mTn with our in house script is introduced in the amplification steps before sequencing on MiSeq. (B) Profile of amplicon 10_01 for a sample compound heterozygous for (TG)11T5 and (TG)11T7. (C) Profile of amplicon 10_01 for a sample homozygous for (TG)11T7. (D) Amplicons targeting exons 19 of CFTR and controls regions 7, 8 and 9 on chromosome 6 and 20. Amplicon 19_01 also exhibit a slippage which may be related to the presence of a HP stretch of 13 A on the amplicon that generates a high rate of error of the polymerase. This stretch is located 93 nucleotides upstream of exon 19 and was excluded of the ROIs because it generated a high rate of false positive InDels. This support the hypothesis that a significant proportion of the sequencing errors observed in repetitive regions is introduced by PCRs independently of the NGS platform used. (DOC) [file pone.0149426.s002.doc]
