# Supplementary material for: Applicability and Efficiency of NGS in Routine Diagnosis: In-Depth Performance Analysis of a Complete Workflow for CFTR Mutation Analysis
Source: PLoS One. 2016 Feb 22;11(2):e0149426. doi: 10.1371/journal.pone.0149426 (PMC4762772; doi:10.1371/journal.pone.0149426)
Supplement: S1 Table — Samples without pathogenic variants are not represented in this table. Patients with the “Atypical CF” phenotype were referred in the context of respiratory symptoms (e.g. nasal polyposis, chronic rhino-sinusitis, chronic cough) or digestive manifestations (malabsorption) and present intermediary or positive sweat test values. Patients with the “Carrier” phenotype do not present any clinical signs evocative of CF or CFTR-RD (healthy parents of a foetus with hyperechogenic bowels are included in this group). 1 Healthy father of a CF child. 2 This patient was refered because of positive family history and is fertile and asymptomatic. (DOC) [file pone.0149426.s003.doc]

**S1 Table : Genotypes identified in the performance monitoring phase (phase 2)**

| **Patient** | **Genotype (cDNAposition)** | **Genotype (Legacy names)** | **Phenotype** |
| --- | --- | --- | --- |
| P-001 | c.472A>G | S158G | Atypical CF (Respiratory Symptoms) |
| P-002 | c.1210-34TG[12]T[5] | (TG)12T5 | Carrier |
| P-003 | c.2900T>C | L967S | Carrier |
| P-006 | c.1210-34TG[12]T[5] | (TG)12T5 | Carrier |
| P-016 | c.508C>T | R170C | Carrier |
| P-018 | c.1210-34TG[12]T[5] | (TG)12T5 | Carrier |
| P-019 | c.1210-34TG[12]T[5] | (TG)12T5 | Carrier |
| P-020 | c.1521_1523del | F508del | Pancreatitis |
| P-021 | c.674G>A | C225Y | Carrier |
| P-022 | c.674G>A | C225Y | Carrier |
| P-023 | c.2450G>T | G817V | Carrier |
| P-026 | c.[1130dup];[3909C>G] | 1259insA/N1303K | CF |
| P-044 | c.1521_1523del | F508del | Pancreatitis |
| P-052 | c.[1327G>T;1727G>C;2002C>T] | D443Y;G576A;R668C | Atypical CF (Respiratory Symptoms) |
| P-055 | c.1210-34TG[11]T[5] | (TG)11T5 | Carrier |
| P-059 | c.1727G>C | G576A | Carrier |
| P-061 | c.[1210-34TG[12]T[5]];[1521_1523del] | (TG)12T5/F508del | CBAVD |
| P-062 | c.1210-34TG[11]T[5] | (TG)11T5 | Carrier |
| P-065 | c.1727G>C | G576A | Pancreatitis |
| P-068 | c.[1521_1523del];[1521_1523del] | F508del/F508del | CF |
| P-070 | c.2735C>G | S912W | Pancreatitis |
| P-074 | c.[350G>A](;)[3415A>G] | R117H/I1139V | Pancreatitis |
| P-076 | c.1521_1523del | F508del | Pancreatitis |
| P-079 | c.3705T>G | S1235R | Atypical CF (Respiratory Symptoms) |
| P-080 | c.1521_1523del | F508del | Carrier |
| P-084 | c.1521_1523del | F508del | Atypical CF (Digestive Symptoms) |
| P-086 | c.2210C>T | S737F | Carrier |
| P-087 | c.2210C>T | S737F | Fetal bowel hyperechogenicity |
| P-090 | c.[350G>A](;)[2490+4_2490+7del] | R117H/2622+4delGGTA | Bronchiectasis |
| P-091 | c.3935A>G | D1312G | Carrier |
| P-103 | c.[1210-34TG[11]T[5];1684G>A] | V526I;(TG)11T5 | Carrier |
| P-122 | c.1210-34TG[11]T[5] | (TG)11T5 | Atypical CF (Respiratory Symptoms) |
| P-126 | c.[350G>A];[1364C>A] | R117H/A455E | Bronchiectasis |
| P-129 | c.1210-34TG[12]T[5] | (TG)12T5 | Bronchiectasis |
| P-141 | c.3705T>G | S1235R | Carrier |
| P-142 | c.3705T>G | S1235R | Carrier |
| P-143 | c.3705T>G | S1235R | Fetal bowel hyperechogenicity |
| P-149 | c.3208C>T | R1070W | Pancreatitis |
| P-150 | c.3485G>T | R1162L | Atypical CF (Respiratory Symptoms) |
| P-151 | c.1210-34TG[11]T[5] | (TG)11T5 | Carrier |
| P-161 | c.3322G>C | V1108L | Carrier |
| P-165 | c.3209G>A | R1070Q | Carrier |
| P-170 | c.[2039del];[1210-34TG[11]T[5]] | (TG)11T5/2171delC | Carrier^1^ |
| P-173 | c.1210-34TG[11]T[5] | (TG)11T5 | Carrier |
| P-174 | c.1210-34TG[12]T[5] | (TG)12T5 | Pancreatitis |
| P-177 | c.650G>A | E217G | Carrier |
| P-179 | c.91C>T | R31C | Carrier |
| P-180 | c.721G>T | G241W | Carrier |
| P-187 | c.1117-5A>G | 1249-5A>G | Carrier |
| P-192 | c.[870‑1113_870‑1110del];[1521_1523del] | 1002‑1113delGAAT/F508del | CF |
| P-200 | c.1521_1523del | F508del | Pancreatitis |
| P-205 | c.1210-34TG[11]T[5] | (TG)11T5 | Pancreatitis |
| P-207 | c.1210-34TG[11]T[5] | (TG)11T5 | Carrier |
| P-208 | c.[220C>T;3808G>A] | R74W;D1270N | Pancreatitis |
| P-211 | c.1210-34TG[12]T[5] | TG(12)T5 | Carrier |
| P-214 | c.3485G>T | R1162L | Carrier |
| P-218 | c.1210-34TG[11]T[5] | (TG)11T5 | Carrier |
| P-219 | c.[1696G>A];[2743G>C] | A566T/V915L | Carrier^2^ |
| P-226 | c.4243-5C>T | 4375-5C>T | Pancreatitis |
| P-227 | c.1210-34TG[11]T[5] | (TG)11T5 | Atypical CF (Respiratory Symptoms) |
| P-229 | c.1521_1523del | F508del | Pancreatitis |
| P-231 | c.[870‑1113_870‑1110del];[1210-34TG[12]T[5]] | (TG)12T5/delGAAT | CBAVD |
| P-232 | c.350G>A | R117H | CBAVD |
| P-234 | c.1210-34TG[12]T[5] | (TG)12T5 | CBAVD |
| P-240 | c.[1624G>T];[1210-34TG[11]T[5]] | (TG)11T5/G542X | CBAVD |
| P-244 | c.[1727G>C;2002C>T] | G576A;R668C | Carrier |
| P-247 | c.[1364C>A];[1521_1523del] | F508del/A455E | CBAVD |
| P-254 | c.2991G>C | L997F | Carrier |
| P-256 | c.[1393-1G>A];[4433C>G] | 1525-1G>A/T1478R | CBAVD |
